# microRNA-106a modulates cisplatin sensitivity by targeting PDCD4 in human ovarian cancer cells

**DOI:** 10.3892/ol.2013.1644

**Published:** 2013-10-29

**Authors:** HAO LI, HAIYUAN XU, HUILING SHEN, HAO LI

**Affiliations:** 1 Department of Clinical Laboratory Medicine, Xiangyang Hospital of Hubei University of Medicine, Xiangyang, Hubei 441000, P.R. China; 2 Department of Oncology, The Affiliated People’s Hospital, Jiangsu University, Zhenjiang, Jiangsu 212001, P.R. China; 3 Department of Central Laboratory, The Fourth Affiliated Hospital, Jiangsu University, Zhenjiang, Jiangsu 212001, P.R. China

**Keywords:** microRNA-106a, drug resistance, PDCD4, ovarian cancer

## Abstract

microRNAs (miRNAs/miRs) are a cluster of short non-protein coding RNAs that negatively regulate gene expression, which is involved in fundamental cellular processes, including the response of tumor cells to chemotherapeutic agents. The present study investigated the role of miR-106a in the development of drug resistance in ovarian cancer cells. The expression of miR-106a in the ovarian cancer OVCAR3 cell line and the cisplatin (CDDP)-resistant ovarian cancer OVCAR3/CIS cell line was detected using stem-loop quantitative (q)PCR. The OVCAR3 and OVCAR3/CIS cells were transfected with mimics or inhibitors of miR-106a or with negative control (NC) RNA using lipofectamine 2000. Luciferase reporter assays were used to determine whether PDCD4 was a direct target of miR-106a in the OVCAR3 cells. The expression levels of the PDCD4 proteins were assessed using qRT-PCR and western blotting, respectively. Drug sensitivity was analyzed using a 3-(4,5-dimethylthiazol-2-yl)-2,5-diphenyltetrazolium bromide assay, while apoptosis was determined by fluorescence-activating cell sorting analysis. The expression levels of miR-106a were upregulated in the CDDP-resistant ovarian cancer OVCAR3/CIS cell line compared with the parental OVCAR3 cell line. However, the PDCD4 protein levels were decreased in the OVCAR3/CIS cells compared with the OVCAR3 cells. The luciferase reporter assays revealed that PDCD4 was a direct miR-106a target in the OVCAR3 cells. Transfection of the OVCAR3/CIS cells with inhibitors of miR-106a enhanced the sensitivity of the OVCAR3/CIS cells to CDDP and increased CDDP-induced apoptosis. The expression of the PDCD4 protein and the sensitivity to CDDP was decreased in the OVCAR3 cells that were transfected with the mimics of miR-106a. The knockdown of PDCD4 expression using PDCD4-specific siRNAs in the OVCAR3 cells demonstrated that PDCD4 is a key signaling molecule in OVCAR3 cell CDDP-induced resistance. miR-106a may be involved in the development of drug resistance and the regulation of PDCD4 expression, at least in part, by modulating CDDP-induced apoptosis in ovarian cancer cells.

## Introduction

Ovarian cancer is the leading cause of mortality from gynecological malignancy and the fifth most common cause of cancer-related mortality in females (1). Currently the standard treatment for advanced-stage ovarian cancer is primary cytoreductive surgery, followed by platinum and paclitaxel combination chemotherapy. Although there have been improvements, the long-term survival rate remains poor due to the emergence of drug resistance. The majority of ovarian cancer patients who are initially sensitive to chemotherapeutic agents will eventually relapse, and in a number of cases, acquired resistance leaves no available curative treatments (2). Emerging evidence indicates that a deregulated apoptosis pathway is a major contributor to tumor initiation and the progression and development of acquired resistance to anticancer therapies (3).

microRNAs (miRNAs/miRs) are a class of non-coding RNAs that regulate gene expression (4) by repressing mRNA translation or cleaving target mRNA. miRNAs play a role in growth control, and an association between miRNAs and cancer is anticipated. Furthermore, miRNAs that are involved in specific networks, including apoptosis, proliferation or receptor-driven pathways, may affect the response to targeted therapies or to chemotherapy. miRNAs are differentially expressed in chemosensitive and chemoresistant cells (5). miR-98, -21 and -125b have been shown to potentiate chemoresistance (6–8). Sorrentino *et al* investigated the role of miRNAs in drug-resistant ovarian cancer cells (9). miR-125b was shown to be downregulated in the A2780/TAX cells and upregulated in the other resistant cell lines. Yang *et al* investigated miRNA expression profiles in cisplatin (CDDP)-resistant ovarian cancer cells and identified that miR-106a was upregulated in CDDP-resistant ovarian cancer cells (10). However, to the best of our knowledge, there have been no studies with regard to the mechanism of miR-106a modulating the sensitivity of ovarian cancer cells to CDDP.

The present study investigated miR-106a expression in CDDP-resistant ovarian cancer cells and the effect of miR-106a downregulation on CDDP chemosensitivity in an ovarian cancer cell line by inducing apoptosis enhancement. miR-106a may be used as a valid therapeutic target in strategies that employ novel multimodality therapies for patients with ovarian cancer.

## Materials and methods

### Cell culture

The human ovarian cancer OVCAR3 cell lines were purchased from the Shanghai Cell Bank of the Chinese Academy of Sciences (Shanghai, China). The CDDP-resistant ovarian cancer OVCAR3/CIS cell line was induced using progressive concentrations of CDDP as described previously (11). The OVCAR3 and CDDP-resistant OVCAR3/CIS cell lines were cultured in PRMI-1640 medium (Gibco, Carlsbad, CA, USA) supplemented with 10% fetal bovine serum (FBS; Gibco), 100 U/ml penicillin and 100 μg/ml streptomycin in a humidified incubator with 5% CO_2_ at 37°C. The OVCAR3/CIS cells were alternately fed with medium containing 7.5 μg/ml CDDP and were regularly tested for the maintenance of drug-resistance. The CDDP-resistant cell line was maintained in drug-free medium for 1 week prior to the follow-up experiments.

### Quantitative reverse transcription PCR (qRT-PCR)

To analyze the miR-106a expression levels, RNA was extracted from the cells. The stem-loop qRT-PCR assay was used to quantify the miRNA expression levels as described previously (12). The qRT-PCR primers were as follows: miR-106a RT primer, 5′-GTCGTATCCAGTGCAGGGTCCGAGGTATTCGCACTGGATACGACCTACCT-3′; miR-106a PCR primer sense, 5′-CGCAAAAGTGCTTACAGTGCA-3′ and antisense, 5′-GTGCAGGGTCCGAGGT-3′; U6 RT primer, 5-CTCAACTGGTGTCGTGGAGTCGGCAA TTCAGTTGAGGGGACAAA-3′; and U6 PCR primer sense, 5′-CTCGCTTCGGCAGCACA-3′ and antisense, 5′-AACGCT TCACGAATTTGCGT-3′. The SYBR Premix Ex Taq™ kit (Takara, Dalian, China) was used according to the manufacturer’s instructions, and qRT-PCR was performed and analyzed using the CFX-96 Real-Time PCR Detection System (Bio-Rad, Hercules, CA, USA). The PCR conditions were 95°C for 3 min, followed by 40 cycles of 95°C for 30 sec, 58°C for 30 sec and 72°C for 30 sec. The expression levels of miR-106a were normalized with reference to the expression levels of U6 snRNA, and the fold changes were calculated by relative quantification (2^−ΔΔCt^) (13). For PDCD4 mRNA detection, qRT-PCR was performed as described previously (14). Each sample was run in triplicate.

### Cell transfection

The cells were seeded in six-well plates to ensure that they would reach 30% confluence the following day. The transfection of the miR-106a mimic, miR-106a antisense oligonucleotide (ASO) or negative control (NC) oligonucleotide was performed using the Lipofectamine 2000 reagent (Invitrogen, Carlsbad, CA, USA) in antibiotic-free Opti-MEM (Invitrogen) according to the manufacturer’s instructions. Following 48 h of transfection, the cells were harvested and processed for further analysis.

### Luciferase reporter assays

The full-length 3′ UTR of PDCD4 was amplified and cloned into the *Xba*1-site of pGL3 (Promega, Madison, WI, USA), checked for orientation, sequenced and named Luc-PDCD4Wt. The site-directed mutagenesis of the miR-21 target site in the PDCD4-3′-UTR was performed using the QuikChange Mutagenesis kit (Stratagene, Heidelberg, Germany), with Luc-PDCD4Wt as a template. For the reporter assays, the OVCAR3 cells were transfected with wild-type (WT) or mutant reporter plasmids and with miR-106a mimics using lipofectamine 2000 (Invitrogen). The reporter assays were performed at 48 h post-transfection using the Dual-luciferase assay-system (Promega) and normalized for transfection efficiency by cotransfected Renilla-luciferase.

### 3-(4,5-dimethylthiazol-2-yl)-2,5-diphenyltetrazolium bromide (MTT) assay

The cells were seeded into 96-well plates at 5×10^3^ cells/well, allowed to grow overnight and then treated with various concentrations of CDDP (QiLu Pharmaceutical, Jinan, China). Following 24 h of treatment, 20 μl 5 mg/ml MTT reagent (Sigma-Aldrich, St. Louis, MO, USA) was added and incubated in the dark for 4 h. The viability of the treated cells was calculated from the average OD 490 values compared with that of the untreated cells. Each treatment was carried out in triplicate.

### Western blot assay

The extraction and detection of the proteins was performed as described previously (15). The protein samples were separated by sodium dodecyl sulfate-polyacrylamide gel electrophoresis and transferred to polyvinylidene difluoride membranes (Millipore, Billerica, MA, USA). The membranes were then blocked with 1% BSA in TBST containing 0.1% Tween-20 for 1 h. The filters were then incubated overnight at 4°C with rabbit mAb against PDCD4, cleaved caspase-3, -8 and -9 (1:1,000; Cell Signaling Technology, Beverly, MA, USA) and mouse mAb GAPDH (1:2,000; Santa Cruz Biotechnology, Inc., Santa Cruz, CA, USA). Immunoblotting was performed by incubating the membranes at 4°C overnight followed by the goat anti-mouse secondary antibodies (Zhongshan Biotechnology Ltd., Co., Beijing, China) conjugated for 1 h at room temperature. Subsequent to washing the membranes, antibody binding was detected using an enhanced chemoluminescence kit (Pierce, Rockford, IL, USA). All western blot experiments were repeated at least three times.

### Knockdown of PDCD4 expression mediated by siRNA

The siRNAs targeting PDCD4 and non-specific NC were purchased from Ambion (Austin, TX, USA). The siRNAs were transfected into the OVCAR3 cells using Lipofectamine 2000 reagent (Invitrogen) according to the manufacturer’s instructions. Following the treatment, the cells were harvested for the subsequent experiments. The experiment was repeated three times.

### Apoptosis assay

To quantify CDDP-induced apoptosis, annexin V/propidium iodide (PI) staining was performed and apoptosis was evaluated using flow cytometry (FCM) analysis. Briefly, following the treatment with PDCD4 siRNA and CDDP, floating and attached cells were collected and subjected to annexin V/PI staining using an annexin V-FITC Apoptosis Detection kit (Keygene, Nanjing, China), according to the manufacturer’s instructions. The resulting fluorescence was measured by FCM using the Becton Dickinson FACSCalibur (Becton Dickinson, Franklin Lakes, NJ, USA).

### Statistical analysis

All the quantitative data were analyzed using Student’s t-tests. All the tests that were performed were two-sided. P<0.05 was considered to indicate a statistically significant difference.

## Results

### Expression of PDCD4 correlates with the cytotoxic activity of CDDP in ovarian cancer cell lines

**Figure 1:**
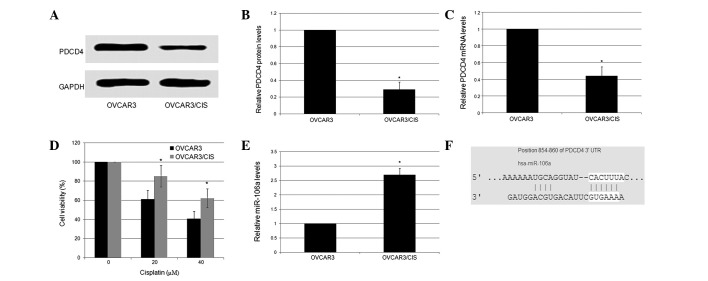
PDCD4 expression correlates with the cytotoxic activity of cisplatin in ovarian cancer cell lines. (A and B) The protein levels of PDCD4 in the OVCAR3 and OVCAR3/CIS cells. (C) The mRNA levels of PDCD4 in the OVCAR3 and OVCAR3/CIS cells. (D) The sensitivity of cisplatin in the OVCAR3 and OVCAR3/CIS cells. (E) The levels of miR-106a in the OVCAR3 and OVCAR3/CIS cells. (F) PDCD4 was predicted to be a potential target of miR-106a. miR, microRNA.

To determine the effect of PDCD4 expression on the sensitivity of ovarian cancer cells to CDDP, the expression of PDCD4 was measured in the OVCAR3 and OVCAR3/CIS cells using qRT-PCR and western blot analysis. As shown in Fig. 1A–C, PDCD4 expression was low in the OVCAR3/CIS cells and high in the OVCAR3 cells. The results from the MTT assays revealed that the OVCAR3 cells with relatively high levels of PDCD4 expression were more sensitive to CDDP compared with the OVCAR3/CIS cells (Fig. 1D). These results indicate that PDCD4 expression may be associated with a high sensitivity to CDDP in ovarian cancer cell lines. The expression levels of miR-106a in the OVCAR3 and OVCAR3/CIS cells were detected using stem-loop qRT-PCR. It was shown that miR-106a had an average 2.63-fold higher expression level in the OVCAR3/CIS cells compared with the OVCAR3 cells (P<0.05; Fig. 1E). These results indicated that miR-106a may play a crucial role in the development of CDDP resistance in epithelial ovarian cancer. TargetScan (http://www.targetscan.org/) was used to computationally predict the targets of miR-106a. The tumor suppressor, PDCD4, was predicted to be one such potential target (Fig. 1F).

### Correlation between miR-106a and CDDP resistance in ovarian cancer cells

**Figure 2:**
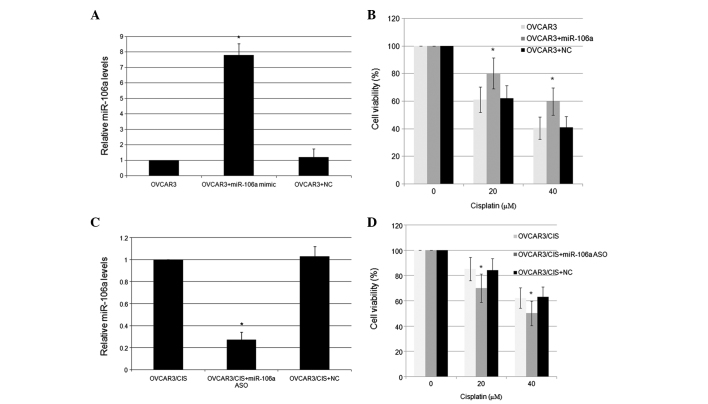
Correlation between miR-106a and cisplatin resistance in ovarian cancer cells. (A) The levels of miR-106a in the OVCAR3 cells that were transfected with the miR-106a mimics or NC. (B) Overexpression of miR-106a was associated with the significantly increased survival rate of the OVCAR3 cells. (C) The levels of miR-106a in the OVCAR3/CIS cells that were tansfected with the miR-106a inhibitors or NC. (D) Downregulation of miR-106a was associated with the significantly increased survival rate of the OVCAR3/CIS cells. miR, microRNA; NC, negative control; ASO, antisense oligonucleotide.

To directly test the correlation between miR-106a and CDDP resistance in the ovarian cancer cells, miR-106a expression was functionally changed using mimics and inhibitors *in vitro,* and subsequently, the resulting alterations of the drug sensitivity were evaluated by the MTT assay. In response to transfection with 100 pmol miR-106a mimics, the expression level of miR-106a in the OVCAR3 cells was increased 7.8-fold compared with the NC (Fig. 2A). The overexpression of miR-106a was associated with the significantly increased survival rate of the OVCAR3 cells (Fig. 2B). The OVCAR3/CIS cells were transfected with either the miR-106a ASO or an NC, and were subsequently incubated with various doses of CDDP. Conversely, transfection with 100 pmol miR-106a inhibitors effectively reduced miR-106a expression and resulted in a significantly lower survival rate in the OVCAR3/CIS cell lines (Fig. 2C and D).

### PDCD4 is a target of miR-106a

**Figure 3:**
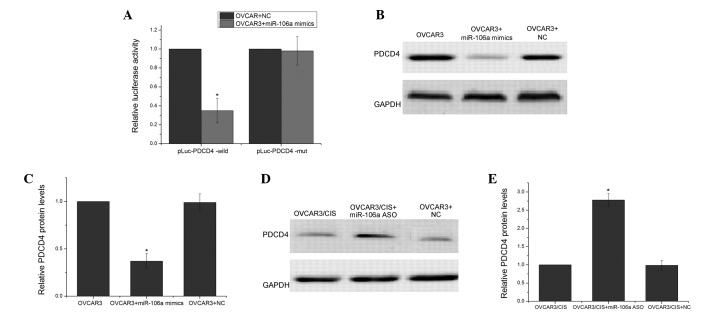
PDCD4 was a target of miR-106a. (A) Luciferase reporter assays confirmed that PDCD4 was a direct miR-106a target in the OVCAR3 cells. (B and C) The expression levels of PDCD4 protein in the OVCAR3/CIS cells that were transfected with the miR-106a inhibitor were significantly increased compared with that in the OVCAR3/CIS cells that were transfected with the control inhibitor. (D and E) The expression levels of PDCD4 protein in the OVCAR3 cells that were transfected with the miR-106a mimic were significantly increased compared with that in the OVCAR3 cells that were transfected with the control inhibitor. miR, microRNA; NC, negative control; ASO, antisense oligonucleotide.

PDCD4 protein expression was significantly downregulated in the OVCAR3/CIS cells compared with the parental OVCAR3 cells. However, it remains unclear whether the downregulation of PDCD4 induced by the overexpression of miR-106a is involved in the resistance of the OVCAR3 cells to CDDP. Computer-aided algorithms were obtained from TargetScan (http://www.targetscan.org/) and the potential binding site of miR-106a (position 854–860) was predicted to be the PDCD4 3′-UTR. As shown in Fig. 3A, transfection of the miR-106a mimics in the OVCAR3 cells with the WT 3′-UTR (pLuc-PDCD4 3′-UTR-wild) vector significantly decreased the luciferase activity compared with the control inhibitor (P<0.05). However, transfection of the miR-106a mimics in the OVCAR3 cells with the mutant 3′-UTR (pLuc-PDCD4 3′-UTR-mut) vector showed no effect on luciferase activity compared with the control inhibitor (P>0.05). Furthermore, the expression levels of the PDCD4 protein in the OVCAR3/CIS cells that were transfected with the miR-106a inhibitor were significantly increased compared with that of the OVCAR3/CIS cells that were transfected with the control inhibitor (P<0.05; Fig. 3B and C). Conversely, the expression levels of the PDCD4 protein in the OVCAR3 cells that were transfected with the miR-106a mimic were significantly increased compared with that of the OVCAR3 cells that were transfected with the control inhibitor (P<0.05; Fig. 3D and E). All these data indicated that PDCD4 was post-transcriptionally regulated by miR-106a in the OVCAR3 cells.

### PDCD4 is a key signaling molecule in induced CDDP resistance in OVCAR3 cells

**Figure 4:**
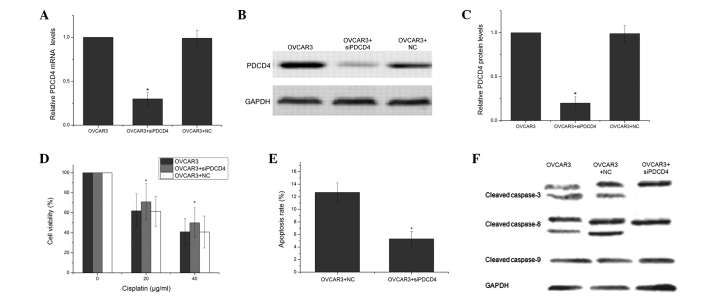
PDCD4 is a key signaling molecule in induced OVCAR3 cell cisplatin resistance. (A) Following the transfection of siPDCD4, the PDCD4 mRNA levels were decreased. (B and C) Following the transfection of siPDCD4, the PDCD4 protein levels were decreased. (D) PDCD4 siRNA significantly increased cell viability when compared with the control cells that were treated with the NC. (E) PDCD4 siRNA decreased the apoptosis induced by cisplatin. (F) PDCD4 siRNAs decreased the expression of cleaved caspase-3 and caspase-8 in the OVCAR3 cells. NC, negative control.

To further confirm the effect of PDCD4 on the chemosensitivity of ovarian cancer cells, PDCD4 expression was knocked down using PDCD4-specific siRNAs in the OVCAR3 cells. As shown in Fig. 4A–C, the PDCD4-specific siRNAs markedly inhibited the expression of the PDCD4 mRNA by 70% and the PDCD4 protein by 80%, whereas the NC had no significant effect on PDCD4 expression. The PDCD4 siRNAs significantly increased cell viability compared with the cells that were treated with the NC (P<0.05; Fig. 4D). This indicated that the downregulation of PDCD4 enhanced the resistance to CDDP. The PDCD4 siRNAs and the scrambled siRNA-transfected OVCAR3 cells were analyzed using FCM to determine cell apoptosis. The PDCD4 siRNAs were identified to decrease the level of CDDP-induced apoptosis in the OVCAR3 cells. (P<0.05; Fig. 4E) To further examine the particular apoptotic pathways by which PDCD4 promotes CDDP-induced apoptosis, the expression of several apoptosis-related proteins was measured. As shown in Fig. 4F, treatment with the PDCD4 siRNAs decreased the expression of cleaved caspase-3 and caspase-8 in the OVCAR3 cells compared with the OVCAR3 cells that were treated with the NC.

## Discussion

Platinum-based combination chemotherapy is the most widely used method in the treatment of ovarian cancer (16). However, due to resistance, the method often fails to cure patients. Therefore, the reversal of platinum resistance in ovarian cancer and increased sensitivity to platinum-based chemotherapy drugs are crucial issues.

miRNAs are a growing class of small, non-coding RNAs that regulate gene expression by targeting mRNAs to cause translational repression and/or degradation. A large number of miRNAs have been identified as deregulated in various types of human malignancy. Increasing evidence indicates that the deregulation of miRNAs has been frequently observed in cell proliferation, differentiation, apoptosis, metastasis and drug resistance (17–20). The mechanisms responsible for the chemotherapy resistance by miRNAs have not been clearly identified. To date, several miRNAs, including miR-451, -21, -214, -23a and -141, have been reported to be involved in the process of CDDP resistance in various tumors (21–23). Based on these findings, Fu *et al*(11) performed global miRNA expression profiling in human ovarian CDDP-resistance and parental cancer cells, and identified that miR-15a, -19a, -21, -204, -93 and -96 were upregulated and that miR-22 and -489 were downregulated. The present study identified that miR-106a was overexpressed 2.7-fold in the CDDP-resistant OVCR3/CIS cells compared with the corresponding CDDP-sensitive parental cell line, and the subsequent qRT-PCR experiment confirmed this result. Knockdown of miR-106a enhanced CDDP chemosensitivity in the CDDP-resistant OVCR3/CDDP cells, while the ectopic expression of miR-106a caused the OVCR3 cells to be resistant to CDDP-induced apoptosis.

Numerous miR-106a targets have been predicted by TargetScan, including PDCD4. The overexpression of PDCD4 has been reported to increase the sensitivity to CDDP and paclitaxel, but not to etoposide or 5-fluorouracil in human prostate cancer PC3 cells (24). Consistent with this finding, the present study demonstrated that PDCD4 is a target of miR-106a and that it plays a role in CDDP resistance in the OVCR3 cell line. Furthermore, knockdown of PDCD4 significantly increased the cell survival rate and had an overall effect that was similar to miR-106a overexpression. To the best of our knowledge, this is the first study to describe an association between miR-106a, PDCD4 expression and drug resistance in CDDP-treated OVCR3 cells. The loss or reduction of PDCD4 expression may be a reason for chemoresistance in ovarian cancer, and the restoration of PDCD4 expression may reverse the resistance of ovarian cancer to chemotherapy. To date, the mechanism by which PDCD4 enhances chemosensitivity remains unclear. Apoptosis in hepatocellular carcinoma cells was induced by PDCD4 through mitochondrial events and the caspase cascade, including increases in cytosolic cytochrome *c* and mitochondrial Bax and a reduction in procaspase-3, -8, and -9. The present results revealed that the combination of PDCD4 with CDDP markedly elevated the expression of cleaved caspase-3 and -8. Taken together, the data indicated that PDCD4 promoted CDDP-induced apoptosis mainly through the death receptor-mediated pathway.

In summary, the present study demonstrated that the enhancement of miR-106a expression contributes to the generation of CDDP-resistant ovarian cancer cells, partly by targeting PDCD4. PDCD4 promoted CDDP-induced apoptosis mainly through the death receptor-mediated pathway. The results provide evidence that miR-106a may potentially be used as a predictor of the chemotherapy response in ovarian cancer and is a promising therapeutic target in the treatment of this disease.
